# Novel multiphoton intravital imaging enables real-time study of *Helicobacter pylori* interaction with neutrophils and macrophages in the mouse stomach

**DOI:** 10.1371/journal.ppat.1012580

**Published:** 2024-09-30

**Authors:** Hellen Ishikawa-Ankerhold, Benjamin Busch, Almke Bader, Daniela Maier-Begandt, Flavio Dionisio, Sukumar Namineni, Mykhailo Vladymyrov, Ute Harrison, Dominic van den Heuvel, Lukas Tomas, Barbara Walzog, Steffen Massberg, Christian Schulz, Rainer Haas

**Affiliations:** 1 Department of Internal Medicine I, LMU University Hospital, Munich, Germany; 2 Institute of Surgical Research at the Walter-Brendel-Centre of Experimental Medicine, LMU University Hospital, Munich, Germany; 3 German Centre for Cardiovascular Research (DZHK), partner site Munich Heart Alliance, Munich, Germany; 4 Chair of Medical Microbiology and Hospital Epidemiology, Max von Pettenkofer Institute, Faculty of Medicine, LMU Munich, Germany; 5 Institute of Cardiovascular Physiology and Pathophysiology, Biomedical Center, Ludwig-Maximilians-Universität München, Munich, Germany; 6 Data Science Lab, Mathematical Institute, University of Bern, Bern, Switzerland; 7 Department of Immunopharmacology, Mannheim Institute for Innate Immunoscience (MI3), Medical Faculty Mannheim, Heidelberg University, Mannheim, Germany; 8 German Center for Infection Research (DZIF), LMU Munich, Germany; University of Illinois, UNITED STATES OF AMERICA

## Abstract

*Helicobacter pylori* (*H*. *pylori*) is a bacterial pathogen that exclusively colonizes the human gastric mucosa and can cause persistent infection. In this process, *H*. *pylori* employs various strategies to avoid recognition by the human immune system. These range from passive defense strategies (e.g., altered LPS or flagellin structures) that prevent recognition by pattern recognition receptors (PRRs) to more active approaches, such as inhibition of IL-2 secretion and proliferation of T cells via VacA. Despite the growing evidence that *H*. *pylori* actively manipulates the human immune system for its own benefit, the direct interaction of *H*. *pylori* with immune cells in situ is poorly studied. Here, we present a novel intravital imaging model of the murine stomach gastric mucosa and show for the first time the *in situ* recruitment of neutrophils during infection and a direct *H*. *pylori*-macrophage interaction. For this purpose, we applied multiphoton intravital microscopy adapted with live drift correction software (VivoFollow) on LysM-eGFP and CX3CR1-eGFP reporter mice strains in which specific subsets of leukocytes are fluorescently labeled. Multiphoton microscopy is proving to be an excellent tool for characterizing interactions between immune cells and pathogens *in vivo*.

## Author summary

This study focuses on the dynamics of *H*. *pylori* infection and its interaction with myeloid immune cells, using an innovative *in vivo* multiphoton imaging approach to observe real-time events in the infected gastric mucosa. In contrast to conventional methods, this novel technique allows us to dynamically investigate the host immune response. Our results show the spatio-temporal distribution of neutrophils and the internalization of *H*. *pylori* by macrophages over time. Using intravital multiphoton microscopy enhanced with live drift correction software and utilizing LysM-eGFP and CX3CR1-eGFP reporter mice, we established an intravital imaging model for the gastric mucosa of mice. Notably, we observe a significant increase in neutrophil infiltration into the gastric submucosa during early infection, indicating a rapid response of the innate immune system within the gastric crypts. However, in the chronic infection phase, a contrasting decrease in neutrophil influx is seen, indicating an adaptive innate immune response with fewer neutrophils infiltrating the tissue at an increased rate. Importantly, our data suggest that *H*. *pylori* is dependent on macrophages to proliferate and colonize the gastric mucosa during chronic infection.

## Introduction

The gastric pathogen *Helicobacter pylori* causes one of the most common chronic bacterial infections in humans. Diseases such as atrophic gastritis, peptic ulcers, and gastric cancer resulting from *H*. *pylori* infections are the leading causes of infection-related morbidity and mortality worldwide. Thus, it can be estimated that over 800000 new cases of gastric cancer per year are due to *H*. *pylori* infection [1]. Consequently, *H*. *pylori* has been classified as a definitive carcinogen (Class I) by the World Health Organization (WHO) [2]. Importantly, the immune system is unable to clear the infection, resulting in the characteristic gastric inflammation that persists for decades if untreated. Following infection, *H*. *pylori* induces activation of inflammatory cytokines, including interleukin-8 (IL-8) [3]. Production and secretion of IL-8 promotes neutrophil activation and monocyte recruitment, resulting in mucosal tissue damage. Thus, secretion of IL-8 is the critical mediator of neutrophil migration to sites of infection and requires direct contact of viable *H*. *pylori* with epithelial cells via its *cag*-type IV secretion system (*cag*-T4SS) [4]. Using the *cag*-T4SS, *H*. *pylori* is able to inject its CagA oncoprotein into gastric epithelial cells and various types of immune cells. For this purpose, *H*. *pylori* uses cell surface molecules of the carcinoembryonic antigen-related cell adhesion molecules (CEACAMs) family as host cell receptors to interact with and inject CagA into epithelial cells as well as immune cells, e.g. neutrophils [5,6]. The initial inflammatory response to *H*. *pylori* infection in humans is studied mainly by histopathologic analysis, where a strong neutrophilic response has been observed. After eradication of the bacteria, the inflammatory reaction of the gastric mucosa subsides. It is well known that *H*. *pylori* actively interferes with the host immune system to prevent its elimination. Important factors include secretion of a vacuolating cytotoxin (VacA) that impedes IL-2 secretion by T lymphocytes [7]. In addition, VacA targets phagocytes to prevent proper phagosome maturation, antigen processing, and presentation [8]. Furthermore, the γ-glutamyl transpeptidase GGT contributes critically to *H*. *pylori*’s tolerizing effects on dendritic cells (DCs) [9]. In addition, the bacteria avoid host recognition by Toll-like receptors (TLRs), via binding of human annexin A5 to lipid A of the bacterial LPS preventing TLR4 signaling [10], and via its modulated flagellin subunit *H*. *pylori* avoids recognition via TLR5 [11]. In animal models, such as the conventional C57BL/6 *H*. *pylori* mouse model, the gastric inflammation is generally lower as compared to the human situation. *In vivo* studies to observe and quantify e.g. macrophages and neutrophil infiltration are still poorly reported. Thus, novel approaches need to be developed to study this phenomenon *in vivo* in more detail using appropriate animal models. Here, we developed for the first time *in vivo* imaging of the gastric mucosa in mice to visualize immune cell interactions with *H*. *pylori in situ*. Intravital multiphoton imaging combined with transgenic mouse lines expressing specific fluorescent labels is a powerful method that allows researchers to directly observe cell dynamics and biological processes in living organisms. The principle of multiphoton imaging permits visualization of organs with significant depth and minimal phototoxicity due to infrared laser light excitation, making it an excellent tool for animal model studies. The technique is suitable for real-time observation of cell-cell interactions in organs commonly used in immunology. It also facilitates visualization of cell movement and direct observation of immune cell interactions and dynamics such as intravascular migration and extravasation [12,13]. The influx of neutrophils into the tissue is an early hallmark of a bacterial infection. The major aim of this study is to visualize neutrophil and macrophage recruitment in the first 24 hours and compare it with long-term *H*. *pylori* infection conditions (up to 6 weeks post-infection). Our approach consists of combining intravital multiphoton microscopy with a transgenic mouse line expressing fluorescently labeled immune cells. The immune system recognizes the bacteria and attempts to eliminate them. Some phagocytic cells such as neutrophils can also take up and inactivate *H*. *pylori* [14]. Since a good *in vivo* model to analyze the role of immune cells during *H*. *pylori* infection is still lacking, we have developed an instrument to visualize labeled immune cells and *H*. *pylori in vivo*. By multiphoton microscopy, it is possible to produce three- or four-dimensional, deep tissue images over extended periods of time with minimal phototoxicity.

Generally, intravital imaging of organs localized in the abdominal cavity is quite challenging due to peristalsis, cardiac and respiratory motion, which compromises image quality and complicates quantification of cell tracking and cell-cell interaction *in vivo*. To circumvent these problems, in our multiphoton setup, we implemented a live-drift correction software named VivoFollow, which allows a real-time correction of the tissue drift by determining the offset between two image stacks acquired at different time points and minimize imaging drift during acquisition [15]. Using this approach, we studied neutrophil and macrophage recruitment during acute and chronic *H*. *pylori* infection. Our results showed a rapid influx of neutrophils during acute *H*. *pylori* infection compared to 6 weeks of infection, and an increased number of macrophages in the chronic stage of infection. *H*. *pylori* can be phagocytized by macrophages, and we show an increased number of bacteria inside macrophages in the chronic stage of infection. The depletion of macrophages resulted in a reduction in bacterial growth, suggesting a contribution of macrophage to bacterial proliferation in the later phase of infection. In conclusion, the imaging techniques we deployed prove to be a useful tool for the *in vivo* study of immune cell dynamics and their interaction with *H*. *pylori*.

## Results

### Inner gastric mucosa preparation for multiphoton intravital imaging

**Fig 1:**
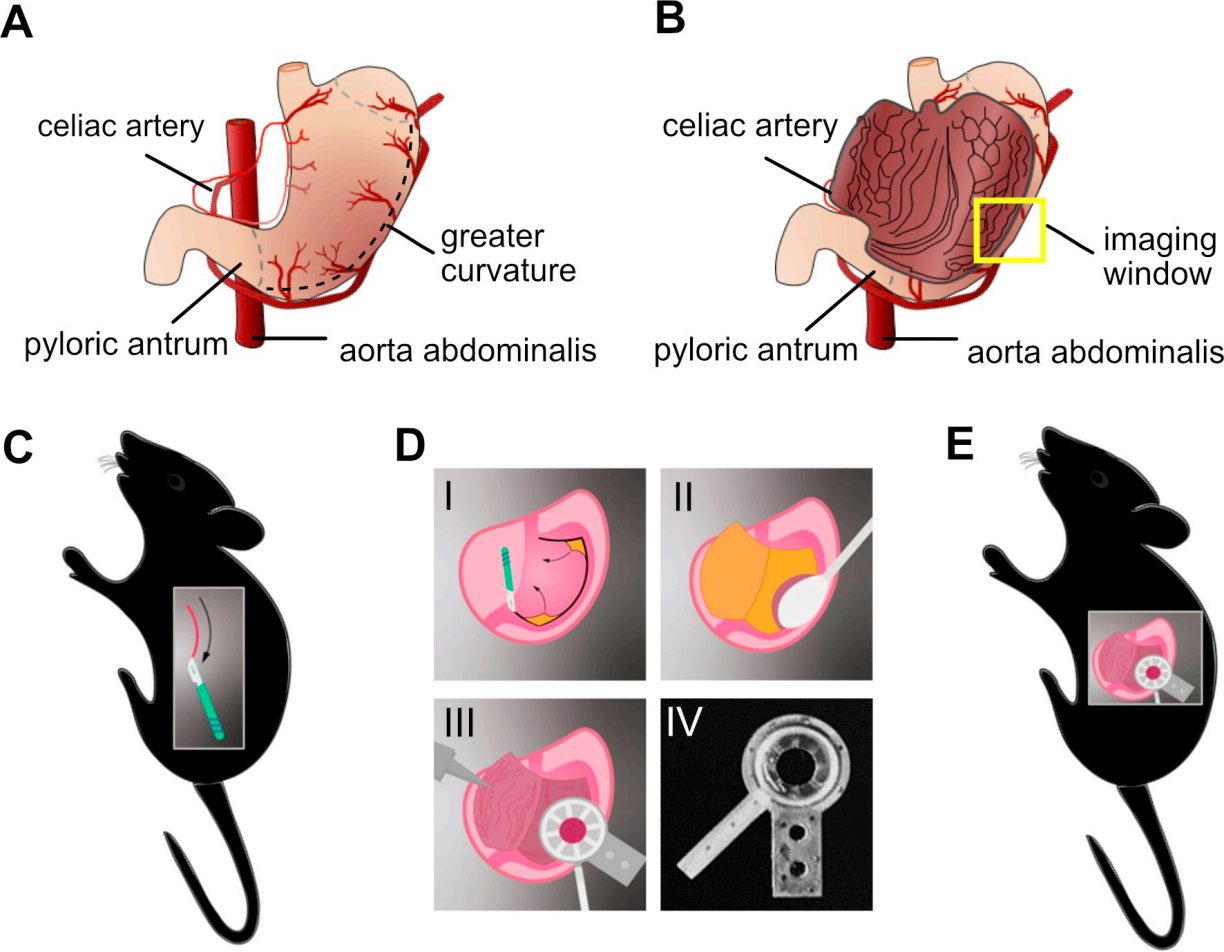
Fixation of murine gastric mucosal tissue for intravital microscopy imaging. (**A**) Cartoon representing the closed (or intact) stomach. The dashed line delineates the area of cutting to open the stomach. (**B**) Open stomach showing the inner gastric mucosa where the yellow square represents the imaging window. (**C**) After sedation, the mouse is placed in the right lateral decubitus position. The stomach is exposed by making an incision of approximately 150 mm across the lateral side. (**D**) Steps demonstrating the gastric mucosa preparation and its fixation by the suction ring device with an imaging window of 8 mm, which was designed, and 3D printed. (**D-I**) After opening the skin, the stomach is opened with a cauterizing scalpel and the gastric mucosa is exposed. (**D-II**) Any remaining gastric contents are carefully removed with a moist cotton swab and irrigation with isotonic solution. Rinsing with isotonic solution, taking care not to injure the gastric mucosa. (**D-III**) The vacuum chamber can now be placed on to the lining of the stomach to permit multiphoton imaging of the gastric mucosa. (**D-IV**) 3D-printed suction ring with an imaging window of 8 mm. (**E**) Overview of the gastric mucosa preparation for multiphoton imaging.

For our initial experiments on visualization of gastric mucosa by multiphoton microscopy, we started with the dissected intact stomach of mice. Due to the thick and rigid muscle layer of the stomach wall, direct microscopy on the intact stomach was very difficult and prevented high resolution imaging of the inner gastric mucosa. To address this issue, the stomach wall was cut laterally to expose the inner gastric mucosa (**Fig 1A and 1B**). Another obstacle we faced is the high peristaltic movement of the organ in the living animal. Therefore, an innovative gastric imaging system was designed in a way to effectively shield the gastric mucosa from the peristaltic movements and the neighboring bowel movement. After opening, the gastric mucosa was carefully cleaned with isotonic solution and food was removed with a moist cotton swab. Next, it was fixed with a suction ring holder attached to a cover slip with an imaging window of 8 mm, for imaging acquisition. The holder was designed and fabricated by the 3D printing technology. The detailed process steps are shown in **Fig 1C–1E**.

### VivoFollow real time drift correction software setup

**Fig 2:**
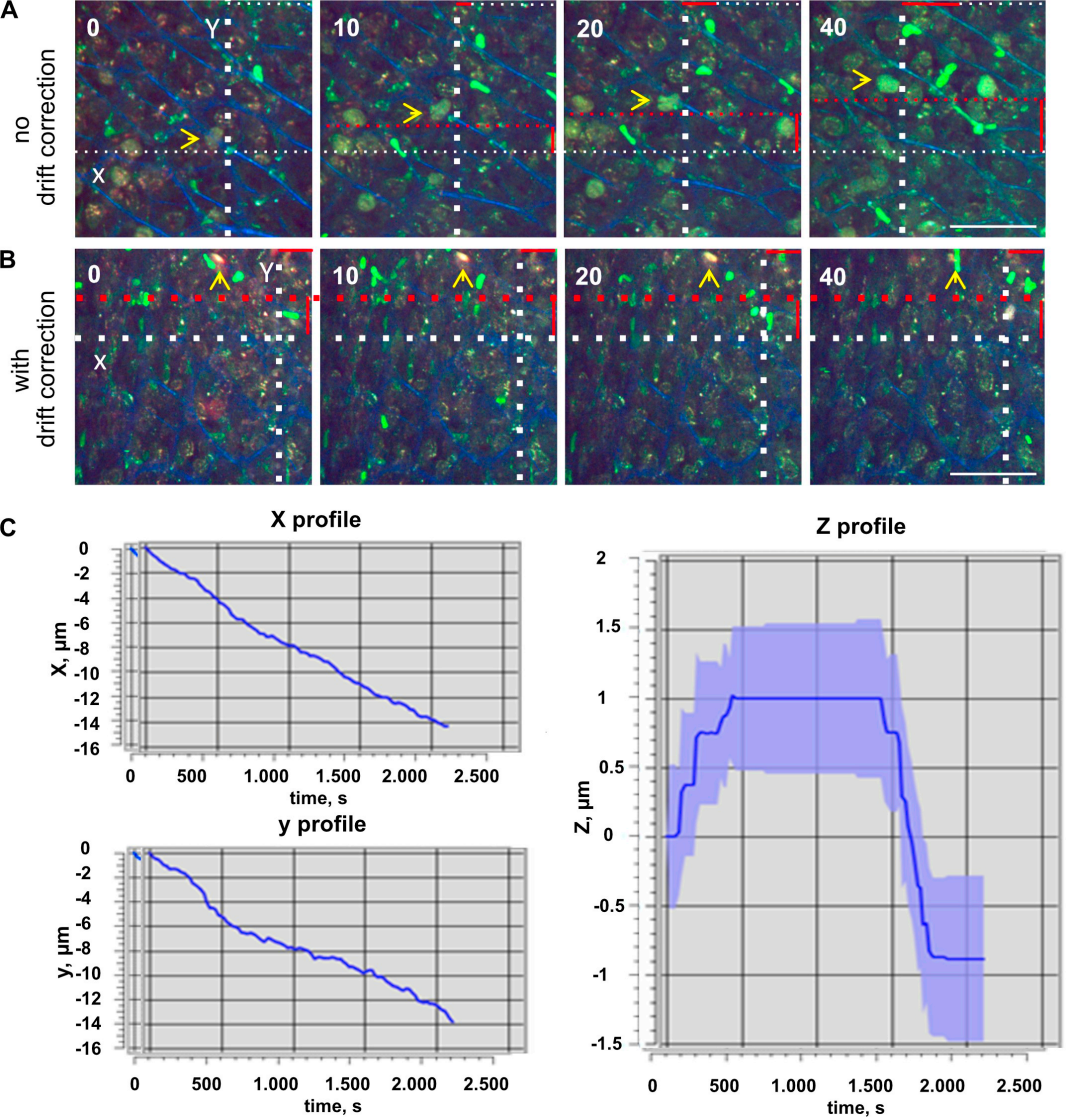
VivoFollow the real time drift correction software. (**A**) Time-lapse sequence imaging of LysM-GFP mouse gastric mucosa showing drift along the X and Y axis. The white dotted lines are for reference; the red dotted lines show the drift, and the red solid lines indicate the magnitude of the shift. The yellow arrow indicates the followed immobilized reference structure showing the imaging drift in (A) and the stable corrected time series in (B). (**B**) Effects of the live drift correction stabilization. There is no major drift from the yellow arrow and white dotted lines serving as references (see also **S1 Video**). Numbers indicate minutes. Scale bars are 50 μm. (**C**) VivoFollow cartoon showing the path of image acquisition and correction in x, y and z, from microscope to GPU-equipped computer.

One of the most challenging tasks in performing intravital microscopy imaging is to avoid excessive vital movements during imaging acquisition, making imaging analyses not possible. A good tissue fixation is essential, however even with a good tissue holding, the longtime imaging acquisition leads to tissue drifting. Therefore, we applied and integrated a real time drift correction software, named VivoFollow, into our system to bypass this issue [15]. A detailed protocol of how to setup image acquisition using the VivoFollow software is described in [16]. The mouse with the fixed gastric mucosa was positioned inside a heated microscope chamber and the mucosa gastric window was placed under a 16x objective. To correct the drift movement during imaging acquisition, the live drift correction software (VivoFollow) was applied to successfully stabilize imaging acquisition *in vivo* (**Fig 2 and S1 Video**).

**Fig 3:**
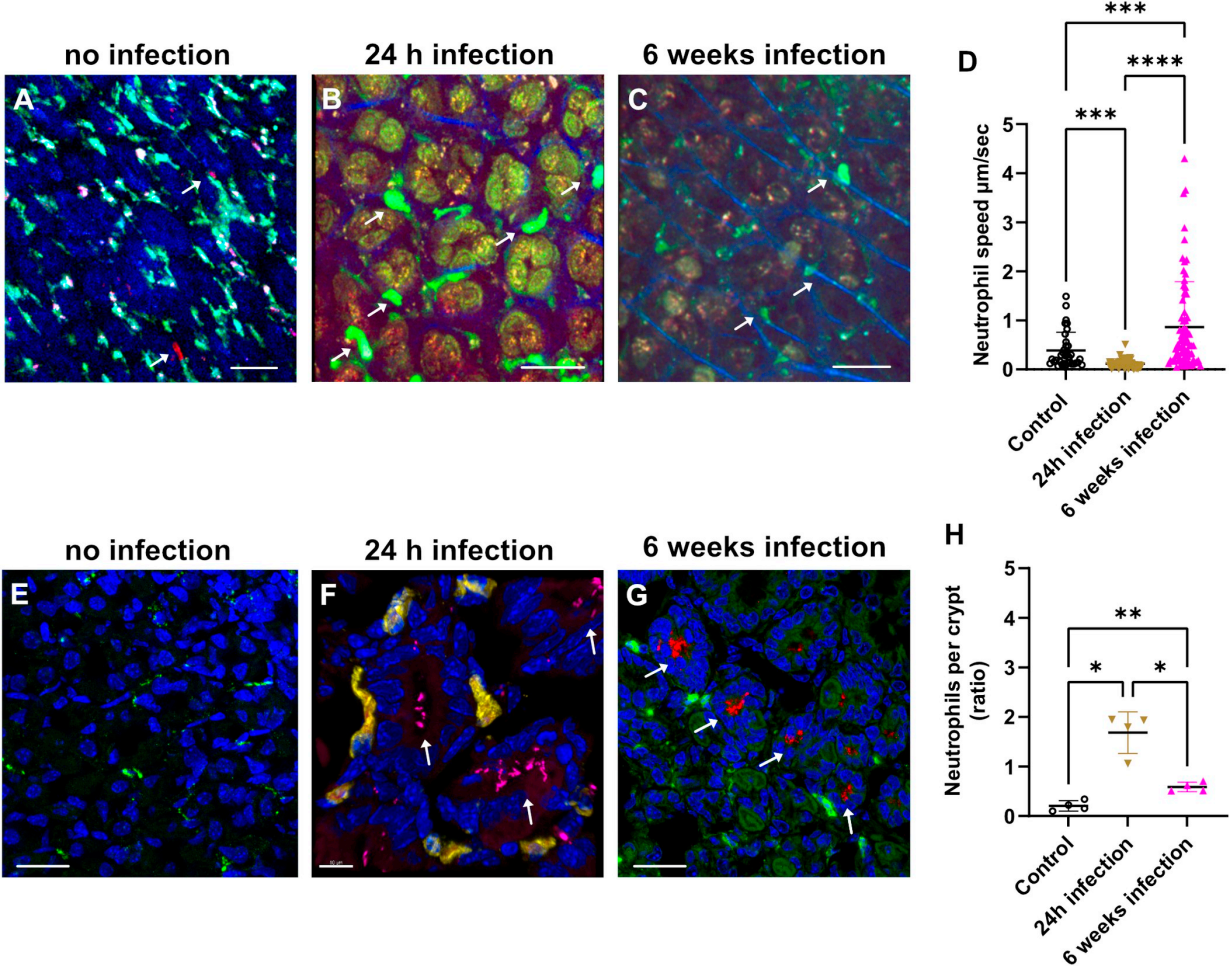
Neutrophil recruitment after *H*. *pylori* infection monitored by multiphoton microscopy *in vivo*. Mice were infected with *H*. *pylori* for 24 hours or 6 weeks. (**A**) Multiphoton 4D time-lapse images of uninfected CX3CR1-GFP^+^ mice, which were injected with anti-Ly6G-PE antibodies to visualize neutrophils in red. (**B**) LysM-eGFP mice show neutrophil recruitment (green) after *H*. *pylori* 24h weeks of infection (red), second harmonic generation (SHG) showing the vessels walls (blue) or 6 weeks after infection in (**C**). (**D**) Histogram showing neutrophil velocity at 24 hours and 6 weeks post-infection. Neutrophils show significantly reduced mobility in the gastric mucosa *in vivo* after the first 24 hours of *H*. *pylori* infection compared to control. After 6 weeks of *H*. *pylori* infection, neutrophil velocity is significantly increased compared to control 24-hours post infection. Immunostaining image of 6 weeks infected gastric mucosal section showing neutrophils stained with anti-Ly6G-488 (green) and *H*. *pylori*-mRFP (red), DAPI (blue). Immunohistology of non-infected gastric mucosa (**E**), scale bar is 20 μm. (**F**) 24h after infection, showing *H*. *pylori* (magenta) localized inside the crypts and neutrophils (yellow). Scale bar is 10 μm. (**G**) 6 weeks after infection, showing *H*. *pylori* (red) located inside the crypts and neutrophils (green), DAPI (nucleus), scale is 20 μm. The arrows indicate the localization of the *H*. *pylori* inside the crypts. (**H**) Quantification of neutrophil influx after 24 hours of *H*. *pylori* infection and decrease after 6 weeks of infection. n = 4 mice. ****p<0.0001, ***p<0.001, **p<0.01, *p<0.05, or not significant (ns). One way ANOVA t-test. Mean ± S.D.

**Fig 4:**
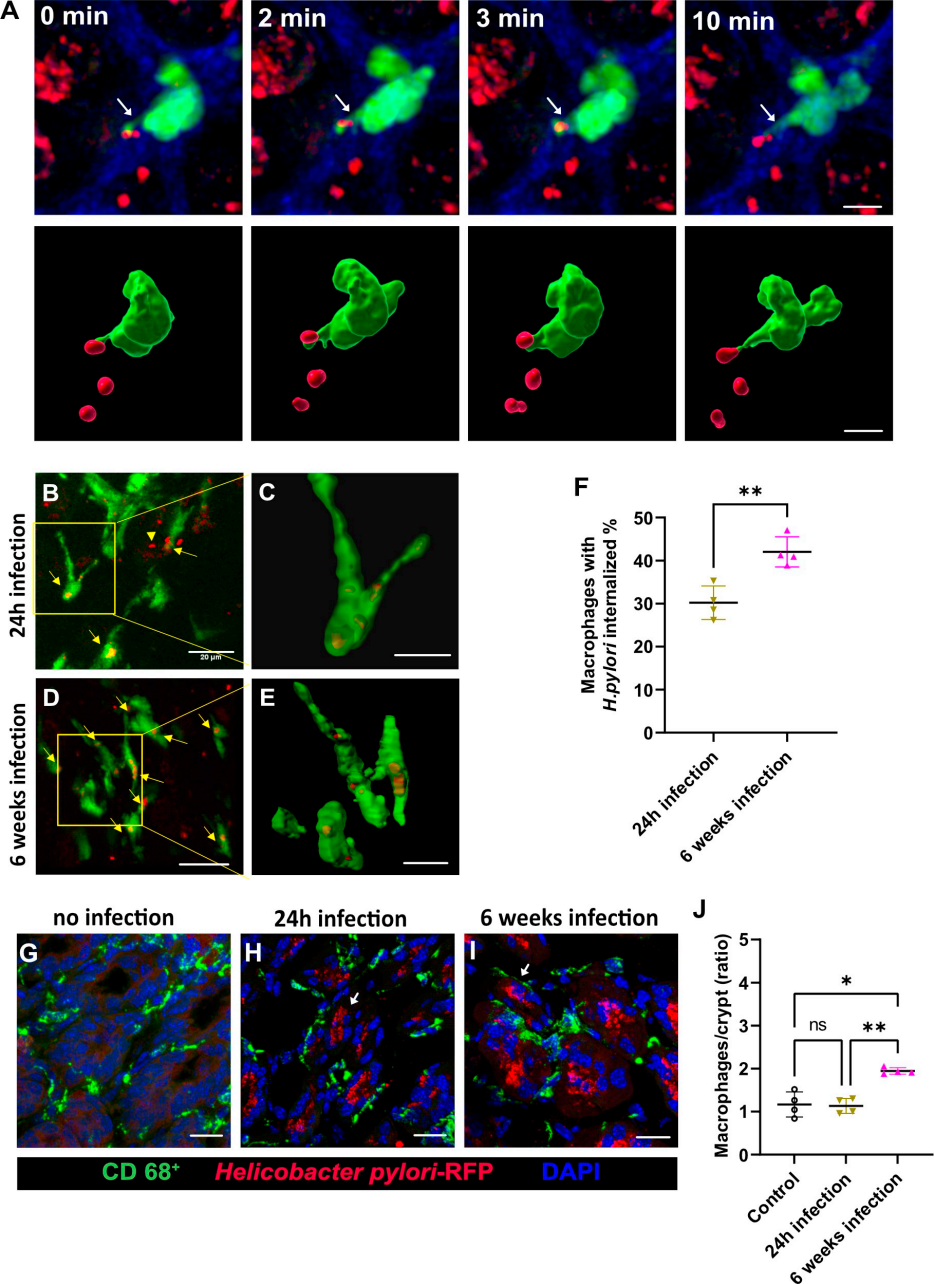
Interaction of macrophages with *H*. *pylori* monitored by multiphoton microscopy *in vivo*. (**A**) Multiphoton imaging of CX3CR1-GFP mouse showing macrophages (green) promoting cytoplasmic extensions in closer contact with *H*. *pylori*, as indicated by the arrows in the upper panel. In the lower panel a 3D image reconstruction of the macrophages (green) and bacteria (red) in a closer proximity. Scale bar is 5 μm. (**B-E**) Multiphoton imaging of CX3CR1-GFP mouse showing *H*. *pylori* (red) localized inside macrophages (green) *in vivo* after 24 hours (**B-C**) or 6 weeks of infection (**D-E**), as indicated by the arrows. (**C and D**) the images represent the zoomed-out macrophages with *H*. *pylori* phagocytized. (**F**) Histogram showing the internalization of *H*. *pylori* by macrophages after 24 hours or 6 weeks of infection. (**G-I**) Immunohistology showing resident macrophages peripherally localized in crypts. Scale bars are 20 μm. (**J**) Quantification of macrophages in gastric mucosa after 24 hours and 6 weeks of *H*. *pylori* infection. n = 4 mice, ns (non-significant difference), ****p<0.0001, ***p<0.001, **p<0.01, *p<0.05. One way ANOVA t test. Mean ± S.D.

From the real-time stable videos acquired, it was possible to analyze the leukocyte dynamics in the gastric mucosa during *H*. *pylori* infection (**Figs 3 and 4**).

### Leukocyte dynamics measurement revealed by multiphoton microscopy *in vivo*

The dynamics of leukocytes during *H*. *pylori* gastric infection of the mouse stomach mucosa can be monitored in real time by using multiphoton microscopy. This high microscopy technology offers a deeper imaging penetration, with less photo-toxicity to the cells. LysM-eGFP mice [17] were infected with *H*. *pylori* for 24h (acute infection) or 6 weeks (chronic infection). The inner gastric mucosa was prepared for multiphoton intravital imaging (**Fig 1**). Gastric mucosa of non-infected, injected with anti-Ly6G-PE antibodies to label neutrophils (**Fig 3A** and **S2 Video**) and LysM-GFP infected mice for 24h (**Fig 3B**, see also **S3 Video**) or 6 weeks (**Fig 3C** and **S4 Video**), were imaged to track neutrophil speed and numbers (**Fig 3D and 3H and S1 Data**). Interestingly, the neutrophil mobility was affected by *H*. *pylori* infection of the gastric mucosa, with significant changes observed at different time points. Specifically, after the initial 24 hours of infection, there was a significant reduction in neutrophil mobility compared to uninfected controls (**Fig 3D**). However, after six weeks of infection, in the chronic infection phase, there was a significant increase in neutrophil velocity compared to both the control and the 24-hour time point. These observations indicate that the effects of *H*. *pylori* infection on neutrophil mobility are time-dependent and may vary during the course of the infection. The quantification of neutrophil influx in response to *H*. *pylori* infection reveals distinct patterns over time. Specifically, there is a significant increase in neutrophil infiltration into the gastric submucosa after the first 24 hours of infection, determined as neutrophils per crypt (**Fig 3E–3H and S2 Data**) representing an early response of the innate immune system to the pathogen localized inside the gastric mucosa crypts (**Fig 3F and 3G** and **S8 Video**). However, after 6 weeks of infection, a marked decrease in neutrophil influx was observed, suggesting that a smaller number of neutrophils is patrolling the tissue with an elevated speed indicating an adaptation of the innate immune system response to the ongoing infection (**Fig 3H**).

### Multiphoton visualization of *H*. *pylori* internalized by macrophages *in vivo* in the murine gastric mucosa

In addition to studying the behavior of neutrophils under conditions of infection over time, the behavior of macrophages in the gastric mucosa was also investigated. The novel multiphoton setup gave us the possibility to visualize the interaction of *H*. *pylori* with macrophages *in vivo* during gastric mucosa infection. We primarily examined resident macrophages labeled with GFP (CX3CR1-GFP) [18]. With this approach, the macrophage cytoplasmic extension in direction to *H*. *pylori* was visualized in the first 24h of infection (**Fig 4A** and **S5 Video**), and the internalization of *H*. *pylori* by the macrophages *in situ* could be visualized after 24h and 6 weeks of infection **(Fig 4B–4E**) and quantified (**Fig 4F and S6 and S7 Videos and S3 Data**). Furthermore, immunohistology revealed resident macrophages localized in crypts in the periphery (**Fig 4G–4I**). Quantification of the data revealed a significant increase in *H*. *pylori* phagocytosis in the chronic phase (6 weeks) as compared to the acute phase (24 hours) (**Fig 4F**). Furthermore, the number of macrophages per crypt also increased significantly from the acute to the chronic phase of infection (**Fig 4J and S4 Data**). These findings suggest that macrophages play a crucial role in the immune response to *H*. *pylori* infection and the study highlights the use of multiphoton microscopy *in vivo* to monitor the interaction between immune cells and pathogens, which could provide a deeper understanding of the immune response to infections.

### Macrophages contribute to *H*. *pylori* proliferation over time

**Fig 5:**
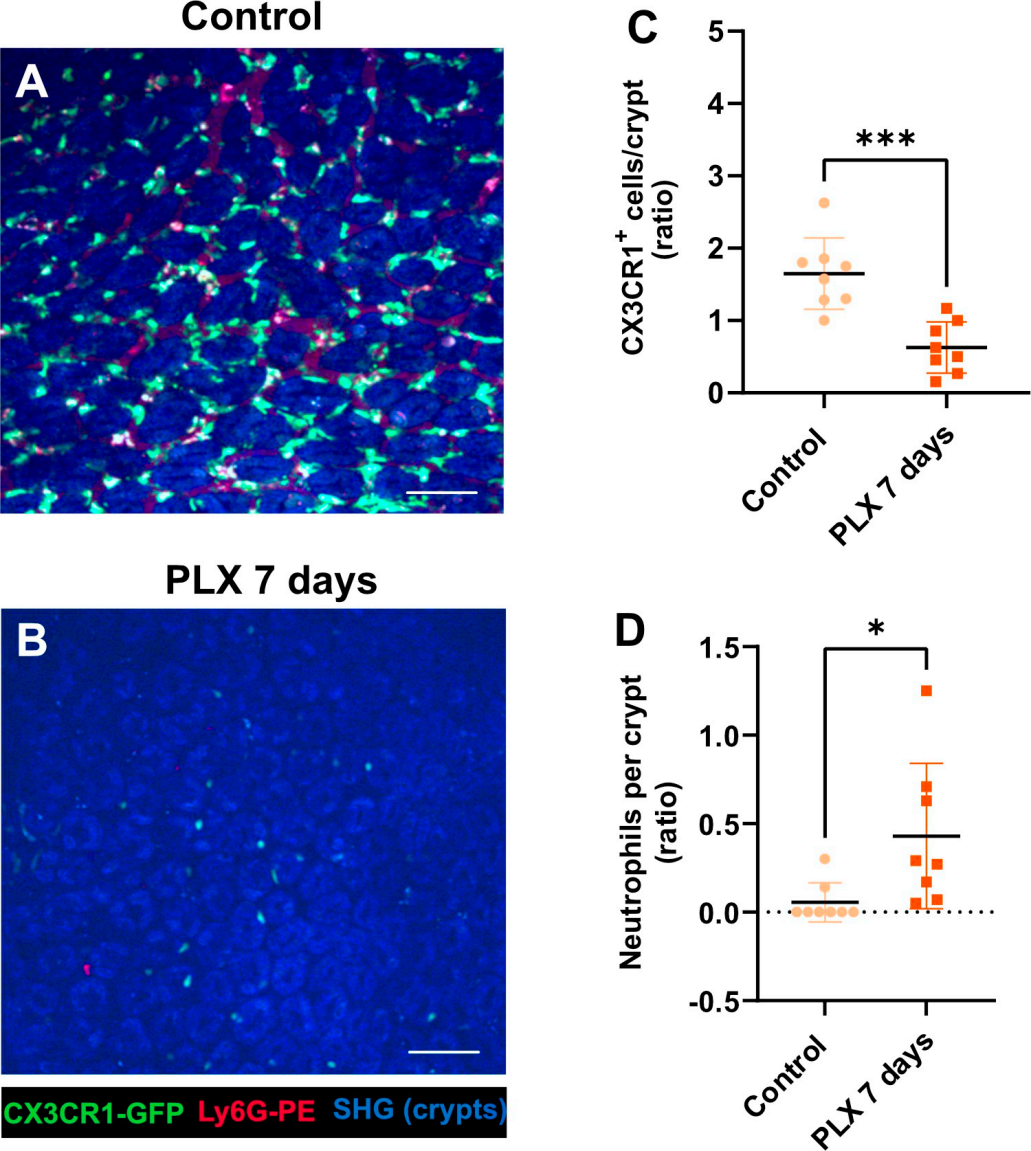
Macrophage depletion and neutrophils response under PLX 5622 treatment. (**A-B**) Multiphoton imaging of CX3CR1-GFP mouse gastric mucosa showing macrophages (green) and neutrophils, labelled by the injection of anti-Ly6G-PE antibodies (red), treated with PLX 5622 chow (**B**) and control chow treated **(A)**, SHG (blue), scale bar is 20 μm. (**C**) Quantification of the CX3CR1-GFP positive cells after 7 days of PLX 5622 treatment. (**D**) Quantification of neutrophils in gastric mucosa after PLX treatment versus untreated (control). n = 8 mice, ns (non-significant difference), ****p<0.0001, ***p<0.001, **p<0.01, *p<0.05. One way ANOVA t-test. Mean ± S.D.

**Fig 6:**
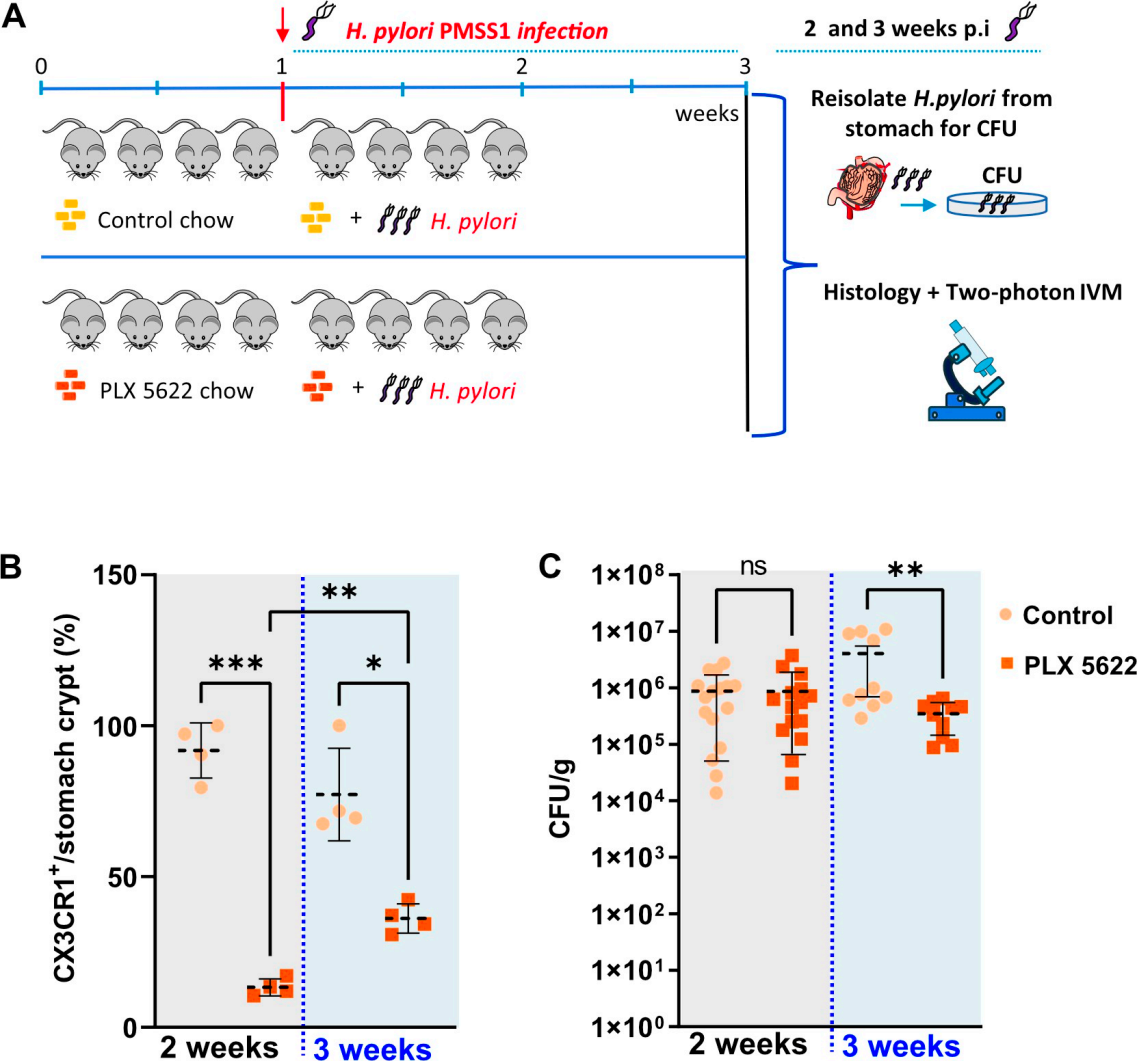
Continuous macrophage depletion is associated with reduced *H*. *pylori* infection. (**A**) Schematic representation of *H*. *pylori* infection (2–3 weeks) and PLX 5622 food treatment (3–4 weeks) to reduce macrophage replication. (**B**) Quantification of CX3CR1+ cells in the gastric mucosa after PLX treatment versus untreated (control) for 2 or 3 weeks of infection. n = 4 mice for each treatment. (**C**) Determination of colony forming units (CFU) of *H*. *pylori* infected CX3CR1+ mice after PLX treatment (PLX 7 days) or untreated (control). ns (non-significant difference), ****p<0.0001, ***p<0.001, **p<0.01, *p<0.05. One way ANOVA t-test. Mean ± S.D. Mouse cartoon by lemmling, sourced from Open Clip Art, licensed under CC0 1.0.

To investigate if macrophages in the gastric mucosa are able to actively interfere with *H*. *pylori* proliferation, e.g. by restricting the number of colonizing bacteria due to phagocytosis, or by facilitating the bacteria proliferation in macrophages, we made use of PLX5622, an inhibitor of the colony-stimulating factor 1 receptor (CSF1R), which was originally used to eliminate microglia from the brain [19]. We show that feeding of mice with PLX5622 chow for 7 days (**Fig 5A–5C and S5 Data**), 2 or 3 weeks (**Fig 6B**), strongly reduces or eliminates resident CX3CR1 macrophages from the stomach mucosa. Furthermore, we show that the decrease in macrophage number was accompanied by an increase in neutrophil counts (**Fig 5D and S6 Data**).

Macrophages contribute to the multiplication of *H*. *pylori* as demonstrated by PLX 5622 treatment and infection for 2–3 weeks (**Fig 6A–6C and S7 Data**). Reducing the number of macrophages on the gastric mucosa by approximately 80% has no significant effect on bacterial colonization efficiency after 2 weeks of infection, but does affect bacterial growth after 3 weeks of infection, as determined by the re-isolation of bacteria (**Fig 6C and S8 Data)**. This indicates that *H*. *pylori* might rely on macrophages to proliferate and colonize the gastric mucosa during chronic infection.

**Fig 7:**
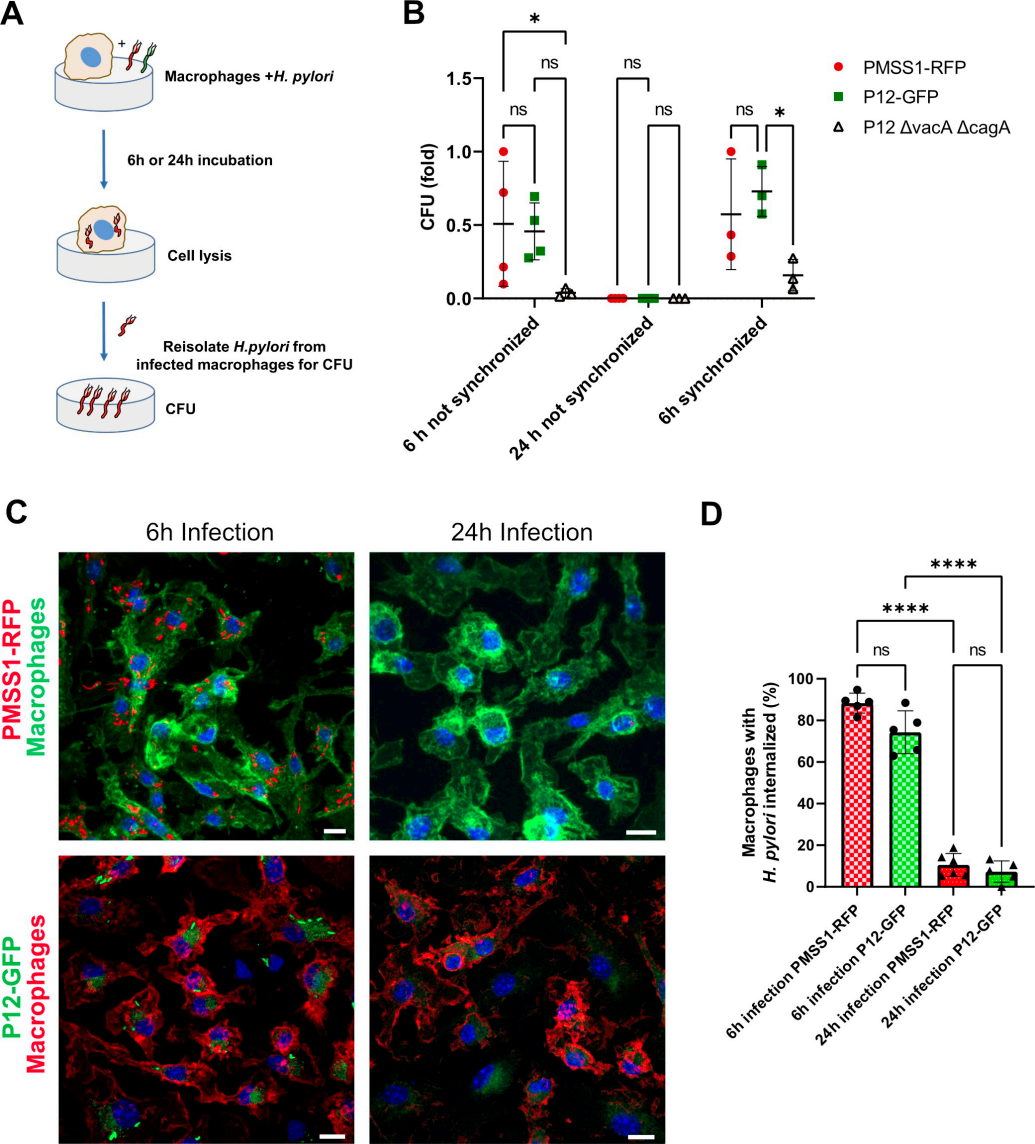
*H*. *pylori* survival in infected macrophages. **(A)** Bone marrow-derived mouse macrophages were incubated with *H*. *pylori* PMSS1-RFP, P12-GFP or P12Δ*vacA*Δ*cagA*. After 6h or 24h infected macrophages were lysed, and *H*. *pylori* were re-isolated for colony forming unit (CFU) assay. **(B)** CFU quantification indicating the bacterial survival after 6h of internationalization by macrophages. **(C)** Infected macrophages were fixed and stained with anti-F4/80-Alexa-488 (green) or Alexa-594 (red) for macrophages, and DAPI for nucleus (blue). Images show *H*. *pylori* internalized by macrophages. Scale bar is 10 μm. **(D)** Quantification of macrophages infected with *H*. *pylori* strains PMSS1-RFP (red) or P12-GFP (green) for 6h or 24h. n = 5–6, ****p<0.0001, ***p<0.001, **p<0.01,*p<0.05, or not significant (ns). Two-way ANOVA t-test. Mean ± S.D.

**Fig 8:**
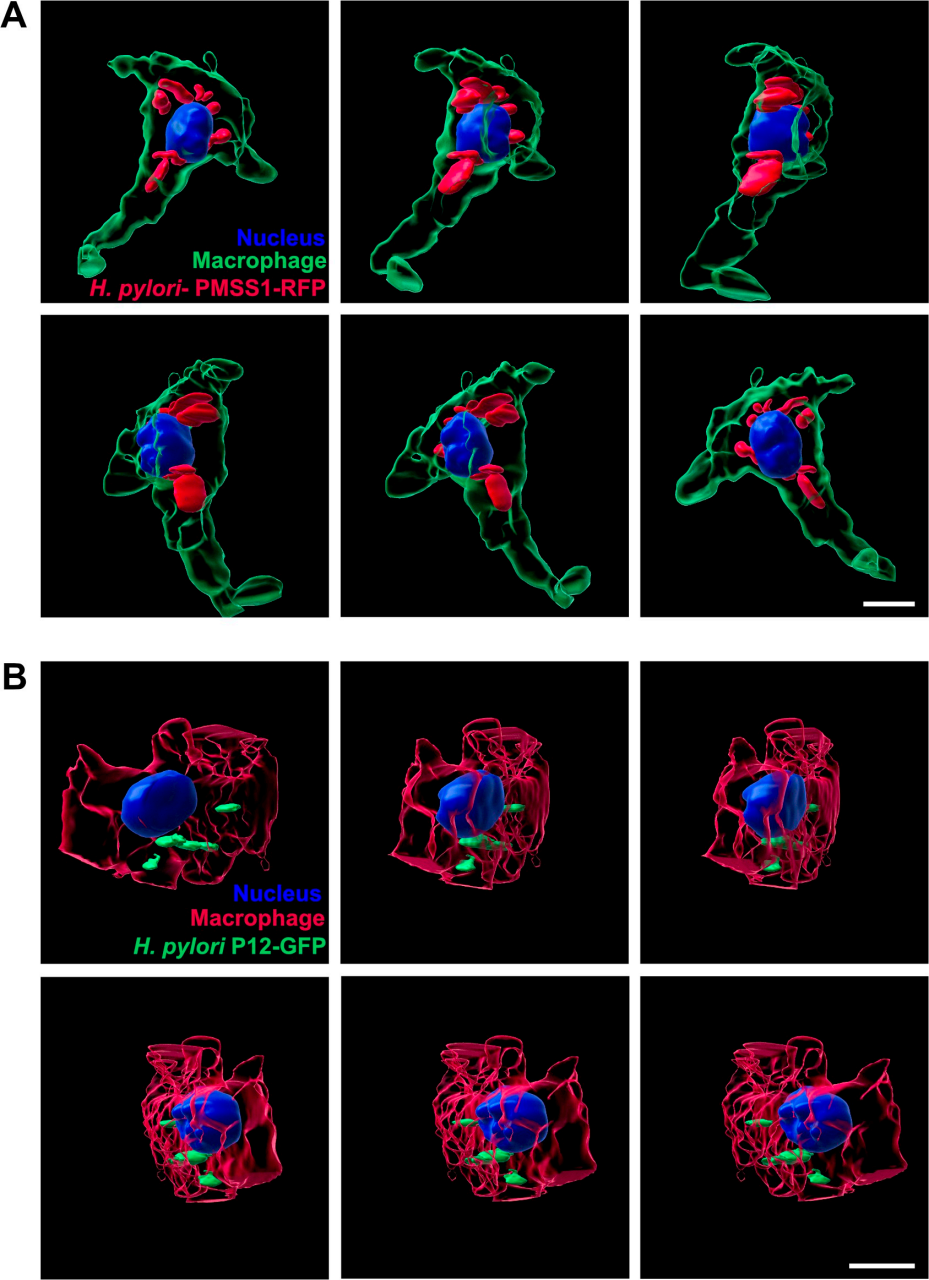
*H*. *pylori* internalization by macrophages. 3D rendering image sequences showing the localization of PMMS1-RFP **(A)** or P12-GFP **(B)** after 6h of infection inside macrophages. The images series are displayed from different angles and were extracted from **S9** and **S10 Videos**. Scale bar is 10μm. DAPI is labelling the nucleus. Anti-F4/80 antibodies were used to label macrophages.

To investigate whether *H*. *pylori* can replicate within macrophages, we conducted a survival assay using isolated primary mouse bone marrow-derived macrophages infected with the bacteria. After incubating the primary macrophages and bacteria for 6 or 24 hours, we lysed the macrophages and re-isolated the bacteria via agar plating to determine bacterial survival **(Fig 7A and 7B)**. We used two *H*. *pylori* strains for the infections: PMSS1-RFP and P12-GFP [10], along with a P12 strain lacking the *vacA* and *cagA* genes (P12Δ*vacA*Δ*cagA*) **(Fig 7B and S9 Data)**, as these virulence factors might influence intracellular survival [20]. Both PMSS1-RFP and P12-GFP strains successfully infected macrophages **(Fig 7C and 7D and S10 Data)**, whereas the P12Δ*vacA*Δ*cagA* mutant showed a very low infection rate **(Fig 7B)**. To ensure this was not due to reduced physical interaction between the mutant strain and macrophages, we included an additional centrifugation step during the 6-hour synchronized infection (see Methods for details), which resulted in better infection, but still at a significantly reduced level, suggesting that the bacteria use an active mechanism for intracellular survival. The localization of the PMSS1-RFP and P12-GFP strains inside macrophages with higher resolution is displayed in **Fig 8** and **S9** and **S10 Videos** and **S1 Fig**.

## Discussion

Our study presents a novel gastric imaging system that was designed to allow for the first time a direct view into the stomach mucosa of the cellular events, such as neutrophil dynamics and *H*. *pylori*–macrophage interaction in an infected animal. The novel system shields the gastric mucosa from peristaltic movements and neighboring bowel movements, allowing for high-resolution imaging of the inner gastric mucosa. In addition, the use of the VivoFollow live drift correction software successfully stabilizes imaging acquisition *in vivo*, enabling analysis of leukocyte dynamics in the gastric mucosa during *H*. *pylori* infection. The combination of these innovative techniques provides a powerful tool for studying the immune response in the gastric mucosa.

Furthermore, our results show that *H*. *pylori* infection affects the mobility of neutrophils at different time points, with a reduction in mobility after 24 hours and an increase after six weeks. It appears that during the acute phase of *H*. *pylori* infection neutrophils arriving in the gastric submucosa lower their speed to monitor the tissue for the bacterial invaders. Immunohistology of the inner gastric mucosa revealed distinct patterns over time. A significant increase in neutrophil infiltration was observed during the first 24 hours of *H*. *pylori* infection, but a marked decrease of neutrophils was seen after 6 weeks, although the bacteria were still present having established the chronic phase of infection. Our earlier data using neutrophils *in vitro* showed that *H*. *pylori* contact strongly enhanced the oxidative burst of neutrophils and stimulated the process of *H*. *pylori* phagocytosis. However, the expression of human CEACAM3/6 on these murine neutrophils allowed the extended survival of *H*. *pylori* within neutrophils [6], suggesting that the conventional *H*. *pylori* mouse model does not adequately mirror the human infection conditions. Nevertheless, the novel *in vivo* multiphoton setup provides first *in vivo* data concerning the adaptation of the innate immune system response to the ongoing *H*. *pylori* infection. Further investigations are needed to fully understand the underlying mechanisms and implications of these findings.

Our study also aimed to investigate the behavior of macrophages in the gastric mucosa during *H*. *pylori* infection using the novel multiphoton setup, which allowed for visualization of the RFP-labeled *H*. *pylori* with resident macrophages labeled with GFP. The experimental data showed an increase in *H*. *pylori* phagocytosis by macrophages in the chronic phase of infection compared to the acute phase, highlighting a crucial role of macrophages in the immune response to this pathogen. Data on phagocytosis of *H*. *pylori* PMSS1-RFP by CX3CR1-GFP macrophages *in vivo* were also previously reported by fluorescence-activated cell sorting (FACS) [21]. Mice that had been neonatally infected and therefore possessed high colonization rates exhibited as adults RFP signals in approximately 4% of the CD11b^+^ myeloid cell population [21]. Our multiphoton microscopy tool revealed the *in vivo* dynamics of macrophage forming protrusions in presence of the bacteria in the early period of infection (**Fig 4A and S5 Video**) and the internalized *H*. *pylori* inside the macrophages in the early and increased number in the later stage of the infection (**Figs 4B–4D and S6 and S7 Videos**). To further understand the functional role of resident macrophages in the control of the bacteria we used the PLX5622 inhibitor of the colony-stimulating factor 1 receptor (CSF1R) to eliminate macrophages from the stomach mucosa. Interestingly, the reduced number of macrophages had a significant effect on bacterial colonization efficiency in the later stage of bacterial proliferation, however, it has no effect in acute infection, suggesting that macrophages, despite their phagocytosis activity shown under *in vivo* conditions, are not able to contribute to the bacterial proliferation or elimination of *H*. *pylori* in acute phase of infection. We show here that elimination of macrophages for long term, causes a reduction of bacterial growth, suggesting that macrophages may serve as a host for *H*. *pylori* proliferation. Earlier work using murine and human macrophage cell lines show that *H*. *pylori* strains expressing the vacuolating cytotoxin interrupt phagosome maturation in macrophages by recruiting and retaining coronin 1A protein [20]. Other studies show that the internalization of *H*. *pylori* by macrophages induces autophagosome formation and these autophagic vesicles are adapted to allow bacterial replication inside macrophages [22]. In another work, it was found that the transient absence of macrophages in the early period of *H*. *pylori* infection reduced the gastric pathology of the disease but did not affect the bacterial load in the stomach [23].

Our primary mouse macrophage infection experiments support the *in vivo* data, indicating that *H. pylori* can infect and survive within primary mouse bone marrow-derived macrophages. The P12 strain lacking *vacA* and *cagA* genes exhibited a significantly lower infection rate, even after improving physical interaction with macrophages, suggesting these virulence factors are crucial for efficient intracellular survival. Our data imply that macrophages may facilitate *H. pylori* proliferation over the course of infection, playing a role in long-term bacterial growth rather than in the acute stage. This mechanism of bacterial residence inside macrophages may contribute to increased resistance to antimicrobial treatment and protection against humoral antibody responses.

## Materials and methods

### Ethics statement

The animal study protocol was approved by the local regulatory agency (Regierung von Oberbayern, Munich, Germany, record number ROB-55.2-2532.Vet_02-18-189 and ROB-55.2-2532.Vet_02-18-183.

### Mice

C57BL/6, CX3CR1-GFP (B6.129P2(Cg)-Cx3cr1tm1Litt/J) (Strain #:005582), LysM-GFP were obtained from The Jackson Laboratory (Boston, MA, USA). The mice were maintained and bred under specific-pathogen free and fed standard mouse diet. All animals undergoing procedures were over 10 weeks old.

### Bacterial strains

*H*. *pylori* strain PMSS1-RFP [21], P12-GFP and P12Δ*vacA*Δ*cagA* [10] were grown on GC agar plates (Oxoid) supplemented with vitamin mix (1%) and horse serum (8%) (serum plates) and cultured in a microaerobic atmosphere (85% N_2_, 10% CO_2_, 5% O_2_) at 37°C.

### Mice infection and bacterial culture for CFU experiments

Mice were fed with PLX 5622 chow (D19101002i, AIN-76A), or control chow (D10001i, AIN-76A) from Research Diets, for 7 consecutive days prior to infection and continued for 2 or 3 weeks after the infection with *H*. *pylori* PMSS1-RFP. Frozen stock of *H*. *pylori* PMSS1-RFP strain was thawed from -80°C and subcultured on GC agar plates (with 1% vitamin mix and 8% horse serum) for five days under microaerophilic conditions (85% N_2_, 10% CO_2_, 5% O_2_). On the sixth day, *H*. *pylori* was taken into liquid culture in Brucella Broth. Mice were infected twice every other day with approximately 6 x 10^7^ CFUs in Brucella Broth using an oral gavage needle. *H*. *pylori* was re-isolated at 2- or 3-weeks post infection from the antrum and corpus following stomach excision and homogenization using a Wheaton homogenizer and selected on DENT plates (Oxoid *H*. *pylori* selective supplement–SR0147E containing Vancomycin, Cefsulodin Trimethoprim, Amphotericin B with Polymyxin B (Simga: P9602), Nalidixin acid (Roche), Bacitracin and 1% vitamin mix and 8% horse serum) for the CFU experiments. The stomach was collected for immunohistology analysis.

### Mice and organ preparation for multiphoton intravital imaging

CX3CR1-GFP or LysM-GFP mice were anesthetized with 5.0 Vol. % Isofluran (cp-pharma, Burgdorf, Germany) and 0.25 l/l oxygen. Mice were injected intraperitoneally with one dose of anesthetic mixture containing Fentanyl (0.05mg/kg body weight, Albrecht GmbH, Aulendorf, Germany), Midazolam (5mg/kg body weight, B. Braun, Melsungen, Hessen, Germany) and Medetomidine (0.5mg/kg body weight, Zoetis, Germany). Mice under narcosis were placed on a warming pad, the eyes were covered with eye cream (Bepanthene) and mice were positioned in right lateral cubic. The skin incision (about 1 cm) was made on the median right lateral position, after skin disinfection. The stomach was pulled out carefully from the abdominal cavity, to allow the visualization of the complete organ. The lateral curvature of the stomach corpus was open using a cautery pen (Bovie Medical Corporation) and the upper part was fleted out as shown in **Fig 1**, to expose the stomach inner mucosa. The food inside the stomach was carefully removed with a wet swab cotton without touching the tissue. The inner gastric mucosa was rinsed with NaCl (0.9%) to remove food residues. The open stomach was fixed by a suction ring, attached with a cover glass of 8 mm diameter, connected with a small vacuum pump, set up with a pressure of 0.2 PA. The mouse was positioned on the microscope stage within an incubator chamber to maintain a constant temperature of 37°C. Ultrasonic gel, acting as an immersion medium, was applied to the cover slip prior to contacting the microscope objective. Gastric mucosa imaging was conducted using a multiphoton LaVision Biotech (Bielefeld, Germany) TrimScope II system connected to an upright Olympus microscope, equipped with a Ti;Sa Chameleon Ultra II laser (Coherent) tunable in the range of 680 to 1080 nm and additionally an optical parametric oscillator (OPO) compact to support the range of 1000 to 1600 nm and a 16 × water immersion objective (numerical aperture 0.8, Nikon). Live images were acquired from 30–40 μm depth, with z-interval of 2–3 μm. 870 nm was used as an excitation wavelength, with 350 × 350 pixels. The signal was detected by Photomultipliers (G6780-20, Hamamatsu Photonics, Hamamatsu, Japan). ImSpector Pro (LaVision) version 275 was used as acquisition software.

### Imaging stabilization and analysis

During multiphoton intravital imaging acquisition, VivoFollow live drift correction software tool was applied to correct for the tissue motion in real-time by maintaining the region of interest in focus over the course of imaging (15–16).

Customized VivoFollow drift correction software (15) was connected and run into the ImSpector Pro version 275. A customized chamber was made to cover the microscope automatic stage, to maintain a stable 37°C warm environment.

### Immunohistology and imaging

Mice were orally infected with *H*. *pylori* strain and after the determinate period of infection, were anesthetized and euthanized by cervical dislocation. The blood was collected by cardiac puncture and the stomachs were perfused with PBS and collected in cold PBS. The stomachs were fixed in 4% PFA overnight followed by overnight infiltration in sucrose at 4°C. Subsequently, the samples were infiltrated in tissue teck and frozen in dry ice, before storage at -80°C. Cryosectioning of the samples with a thickness of 10 μm was performed using a cryotome (Cryostar NX70 from Epredia) in preparation for immunohistological analysis. The sliced tissue was fixed with 4%PFA for 15 min at RT, washed with PBS 3x and blocked with BSA 3% overnight. Next day the primary antibodies were added overnight at 4°C, washed and incubated with the secondary antibodies for 2h at RT. After washing DAPI (1 μg/ml, 1:1000) was added for 30 min, before the tissue mounting with fluorescence mounting medium DAKO. Images were taken using a confocal laser scanning microscope LSM 880 or 980 (Carl Zeiss) with Airyscan super-resolution module, using 63x objective (Nikon) and a 415 x 415 μm or 312 x 312 μm field of views and step size 1.0 μm. Macrophages were labeled with anti-CD68 (clone FA-11, BioRad # MCA1957GA) and neutrophils with anti-ly6G primary antibodies (clone 1A8, Biolegend # 127608).

### Bone marrow-derived macrophages *culture*

Bone marrow-derived macrophages were generated as previously described [24]. Briefly, bone marrow was obtained by flushing murine femur and tibia with ice cold 1x PBS through a 70 μm filter. The suspension was centrifiged with 350 g, 4°C for 5 min, and the cell pellet was resuspended in RPMI1640 medium (Sigma-Aldrich), supplemented with 10% fetal bovine serum (FBS) (BioSell), 1x Pen/Strep (Sigma-Aldrich) and 20 ng/mL macrophage colony-stimulating factor (M-CSF) from (ImmunoTools). Medium was changed every other day. At day 6 of differentiation, macrophages were detached using Accutase (Sigma-Aldrich), and plated in 24-well non-tissue culture treated plates at a density of 2.0x10^5^ macrophages per well.

### Mouse macrophage survival assays

Macrophages were setled into each 24 well (2x10^5^ cells/well) and were washed twice with RPMI medium + 10% fetal calf serum (FCS) to remove antibiotics and kept in 200 μl RPMI + 10% FCS + M-CSF. For macrophage infection, *H*. *pylori* PMSS1-RFP, P12-GFP, and P12Δ*vacA*Δ*cagA* were removed from a serum plate with a sterile cotton swab and resuspended in PBS + 10% FCS. The optical density (OD) at 600 nm was measured to determine the concentration, with an OD of 1 corresponding to 2.5 x 10^8^ bacteria/ml. Bacteria were resuspended in RPMI + 10% FCS + M-CSF to a concentration of 5 x 10^7^ bacteria/ml. 100 μl of this suspension (5 x 10^6^ bacteria, multiplicity of infection: 25) were added per well to the macrophages and incubated at 37°C and 5% CO_2_ for 5 h (6 h time point) or 23 h (24 h time point). Alternatively, to obtain synchronization of infection, plates were centrifuged at 400 g for 6 min at 4°C. Immediately afterwards the cells (*H*. *pylori* infected macrophages) were washed three times with cold RPMI + 10% FCS before addition of pre-warmed RPMI + 10% FCS + M-CSF and incubation at 37°C and 5% CO_2_ for 1 h. In case of syncronized as well as non-synchronized infection assays the cells were washed twice with RPMI + 10% FCS before addition of RPMI + 10% FCS + M-CSF containing 100 μg/ml gentamicin and incubated for 1 h at 37°C and 5% CO_2_. Afterwards, the cells were washed twice with PBS and per well 200 μl 0.1% saponin in PBS was added and incubated for 5 min at room temperature for cell lysis. Different volumes were plated on serum plates and incubated at 37°C and 10% CO_2_ for at least 96 h before colonies were counted.

Infected macrophages were plated on round cover slip of 13 mm diameter, coated with poly-L-lysin (Merck), fixed with 2% PFA for 15 min RT, blocked with 2% BSA for 30 min, followed by adding anti-mouse F4/80-Alexa-488 (Biolegend, #123120) or Alexa 594 (Biolegend, #123140) antibodies, for 1h RT. Washed 3x with PBS, added DAPI (1μg/ml) and mounted with fluorescence mounting medium DAKO.

### Statistical analysis

Visualization and statistical analyses were performed with GraphPad Prism software (San Diego, USA) version 9.0. In all Figs, the data are presented as mean ± SD. An unpaired student‘s t-test was used for comparison between two groups. For comparison of multiple groups with one variable, one-way ANOVA testing was performed. ****p<0.0001, ***p<0.001, **p<0.01, *p<0.05, or not significant (ns).

## Supporting information

S1 Video Multiphoton imaging of gastric mucosa without and with the correction of the VivoFollow software. LysM-GFP mouse was infected with *H*. *pylori*-RFP (red), vessels are visible with the blue color (second harmonic generation; SHG). After 40 min of recording the tissue has drift from the field of view without the drift correction, when the VivoFollow was applied the image was in focus.
(MP4)

S2 Video Multiphoton imaging of the gastric mucosa of no-infected CX3CR1-eGFP mice. There is a reduced number of neutrophils in the non-infected stomach in comparison with the infected mice (S3 Video). CX3CR1-eGFP (macrophages), neutrophils are labelled by the Ly6G-PE antibodies injected (i.v.) into the mice 15 min before imaging. Stomach crypts are labeled blue by the SHG effect.
(MP4)

S3 Video Multiphoton imaging of the LysM-eGFP mice gastric mucosa infected with *H*. *pylori* for 24h. There is an increased number of neutrophils in the 24h infected stomach in comparison with the non-infected mice (S2 Video). LysM-eGFP (neutrophils), *H*. *pylori*-RFP (red) inside the crypts. Vessels are labelled by the SHG.
(AVI)

S4 Video Multiphoton imaging of the LysM-eGFP mice gastric mucosa infected with *H*. *pylori* for 6 weeks. There is an increased speed of the neutrophils in comparison with 24h of infection (see S3 Video for comparison). LysM-eGFP (neutrophils), *H*. *pylori*-RFP (red). Vessels are labelled by the SHG.
(AVI)

S5 Video Intravital imaging of macrophage cytoplasmic extension in closer proximity to *H*. *pylori*. Multiphoton imaging of the CX3CR1-GFP gastric mucosa was infected with *H*. *pylori*-RFP (red) for 24h, macrophage (green), vessels are visible with the blue color (second harmonic generation; SHG), second part of the video show the 3D rendering of macrophage and *H*. *pylori*.
(MP4)

S6 Video Macrophage internalized *H*. *pylori* showed by Multiphoton imaging of gastric mucosa. CX3CR1-GFP mouse infected with *H*. *pylori* for 24h. Macrophages (green) show cytoplasmic extensions and *H*. *pylori* phagocytized (red). The second part of the movie show the imaging 3D reconstruction and rendering.
(MP4)

S7 Video Macrophage internalized *H*. *pylori* showed by Multiphoton imaging of gastric mucosa. CX3CR1-GFP mouse infected with *H*. *pylori* for 6 weeks. Macrophages (green) show cytoplasmic extensions and *H*. *pylori* phagocytized (red). The second part of the movie show the imaging 3D reconstruction and rendering.
(AVI)

S8 Video *H*. *pylori* localization inside the gastric mucosa crypts. LysM-eGFP (yellow) mice gastric mucosa infected with *H*. *pylori* for 24h (magenta). 3D projection shown neutrophils localized at the periphery of the crypts and *H*. *pylori* are located inside the crypts (magenta). Nucleus are displayed in blue.
(AVI)

S9 Video PMSS1-RFP *H*. *pylori* localization inside macrophages. Bone marrow isolated macrophages were co-cultivated with PMSS1-RFP *H*. *pylori* strain (red) and after 6h of incubation the infected macrophages were fixed and labelled with anti-F4/80- Alexa 488 (green). 3D rendering projection shows the internalization of the bacteria by macrophages. Scale bar is 10 μm.
(MP4)

S10 Video P12-GFP *H*. *pylori* localization inside macrophages. Bone marrow isolated macrophages were co-cultivated with P12-GFP *H*. *pylori* strain (green) and after 6h of incubation the infected macrophages were fixed and labelled with anti-F4/80- Alexa 594 (red). 3D rendering projection shows the internalization of the bacteria by macrophages. Scale bar is 10 μm.
(MP4)

S1 Data Corresponding to Fig 3D. (XLSX)

S2 Data Corresponding to Fig 3H. (XLSX)

S3 Data Corresponding to Fig 4F. (XLSX)

S4 Data Corresponding to Fig 4J. (XLSX)

S5 Data Corresponding to Fig 5C. (XLSX)

S6 Data Corresponding to Fig 5D. (XLSX)

S7 Data Corresponding to Fig 6B. (XLSX)

S8 Data Corresponding to Fig 6C. (XLSX)

S9 Data Corresponding to Fig 7B. (XLSX)

S10 Data Corresponding to Fig 7D. (XLSX)

S1 Fig Corresponding to Fig 8. *H*. *pylori* internalized by macrophages. 3D projection image sequences showing the localization of PMMS1-RFP (A) or P12-GFP (B) after 6h of infection inside macrophages. The images series are displayed from different angles representing the raw and unprocessed data used for 3D rendering of Fig 8 and S9 and S10 Videos. Scale bar is 10μm. DAPI is labelling the nucleus. Anti-F4/80 antibodies were used to label macrophages.
(TIFF)
