# Supplementary figures and images for: Novel multiphoton intravital imaging enables real-time study of Helicobacter pylori interaction with neutrophils and macrophages in the mouse stomach
Source: PLoS Pathog. 2024 Sep 30;20(9):e1012580. doi: 10.1371/journal.ppat.1012580 (PMC11478878; doi:10.1371/journal.ppat.1012580)

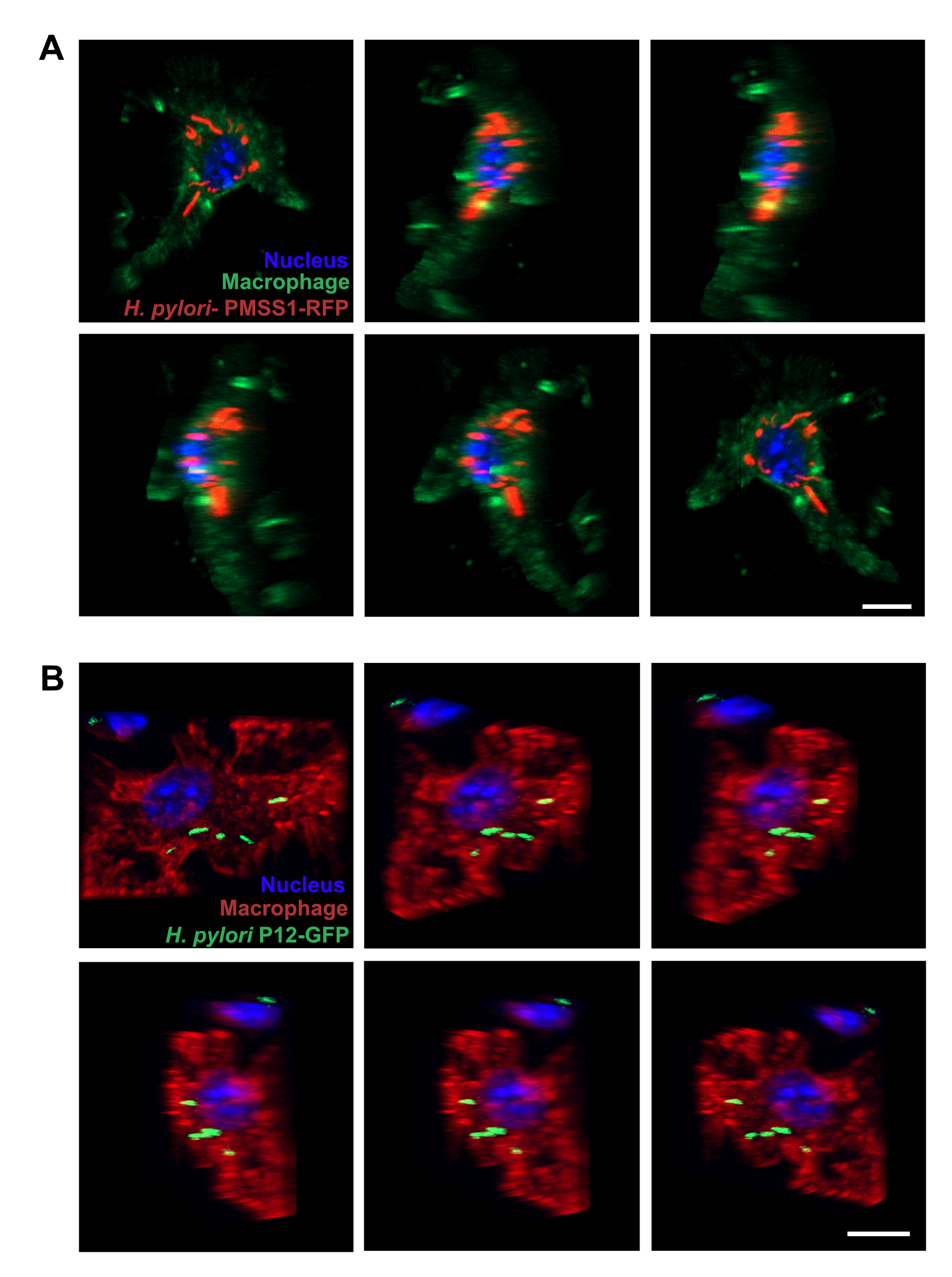

Supplement: S1 Fig — 3D projection image sequences showing the localization of PMMS1-RFP (A) or P12-GFP (B) after 6h of infection inside macrophages. The images series are displayed from different angles representing the raw and unprocessed data used for 3D rendering of Fig 8 and S9 and S10 Videos. Scale bar is 10μm. DAPI is labelling the nucleus. Anti-F4/80 antibodies were used to label macrophages. (TIFF) [file ppat.1012580.s021.tiff]
